# Patient and family centered care: a useful tool to integrate with guidelines!

**DOI:** 10.1186/1824-7288-41-S2-A3

**Published:** 2015-09-30

**Authors:** Raffaele Arigliani, Michele Arigliani, Luciana Parola

**Affiliations:** 1 Primary Care Paediatrician, Scientific Director Italian Medical Research (IMR) SRL, Benevento, Italy; 2 Department of Clinical and Experimental Medical Sciences, Unit of Paediatrics, University-Hospital of Udine, Italy; 3 Unit of Paediatrics and Neonatology, Hospital “G.Fornaroli”, Magenta, Italy

## 

The relationship between the paediatrician, the child and his family is often “paternalistic”:

“I'm the physician in charge, it's just me that knows what's the best for the child's health and I take decisions for the child and his family”.

This approach doesn't consider the autonomy of the patient and his right to have an active role in the decision making process about himself and his health.

Almost every Clinical Guideline recommends to actively involve the patient in the diagnostic-therapeutic work-up but seldom they suggest how to.

The *Patient and Family Centered Care* (PFCC) points out priorities, aims, methods to carry out a clinical approach really focused on the patient and his family and not just on his disease [1].

The *PFCC* highlights the importance of the patient's experience, which will be better if physicians integrate the disease management procedures with standardized “relational procedures” [2], focused on the patient and his family.

According to PFCC the Paediatrician is not interested into taking decisions for the patient (*paternalistic approach*) but he wants to involve him and the family in every step of the diagnostic/therapeutic pathway, turning the relationship into a partnership. The paediatrician should not just treat the disease but also care for the patient and his families, working hard to understand their real needs, wishes and fears, building an empathic relationship in order to make the child and his parents feel accepted, understood and supported.

An increasing number of Scientific Societies recognise the PFCC as an essential element of quality health care [3]. Clinical studies show that the PFCC improves clinical outcomes, entails better compliance to therapies, more satisfaction for health care workers and patients, as well as reduction of costs and legal issues [4].

According to PFCC the health workers have to learn the communication and empathy abilities needed to recognize feelings and wishes of the patients in order to give them answers [5].

Communication is a technical skill, which can be improved through experience and a specific training that should start since the beginning of medical school [6].

In summary the PFCC focuses on the patient, as a person, at 360°, through strategies that are feasible, repeatable, verifiable and continuously evolving, according to evidence.
